# Pneumonic Tularemia in Rabbits Resembles the Human Disease as Illustrated by Radiographic and Hematological Changes after Infection

**DOI:** 10.1371/journal.pone.0024654

**Published:** 2011-09-13

**Authors:** Douglas S. Reed, Le'Kneitah Smith, Tammy Dunsmore, Anita Trichel, Luis A. Ortiz, Kelly Stefano Cole, Eileen Barry

**Affiliations:** 1 Center for Vaccine Research, University of Pittsburgh, Pittsburgh, Pennsylvania, United States of America; 2 Department of Environmental & Occupational Health, University of Pittsburgh, Pittsburgh, Pennsylvania, United States of America; 3 Center for Vaccine Development, University of Maryland, Baltimore, Maryland, United States of America; Wayne State University, United States of America

## Abstract

**Background:**

Pneumonic tularemia is caused by inhalation of the gram negative bacterium, *Francisella tularensis*. Because of concerns that tularemia could be used as a bioterrorism agent, vaccines and therapeutics are urgently needed. Animal models of pneumonic tularemia with a pathophysiology similar to the human disease are needed to evaluate the efficacy of these potential medical countermeasures.

**Principal Findings:**

Rabbits exposed to aerosols containing *Francisella tularensis* strain SCHU S4 developed a rapidly progressive fatal pneumonic disease. Clinical signs became evident on the third day after exposure with development of a fever (>40.5°C) and a sharp decline in both food and water intake. Blood samples collected on day 4 found lymphopenia and a decrease in platelet counts coupled with elevations in erythrocyte sedimentation rate, alanine aminotransferase, cholesterol, granulocytes and monocytes. Radiographs demonstrated the development of pneumonia and abnormalities of intestinal gas consistent with ileus. On average, rabbits were moribund 5.1 days after exposure; no rabbits survived exposure at any dose (190–54,000 cfu). Gross evaluation of tissues taken at necropsy showed evidence of pathology in the lungs, spleen, liver, kidney and intestines. Bacterial counts confirmed bacterial dissemination from the lungs to the liver and spleen.

**Conclusions/Significance:**

The pathophysiology of pneumonic tularemia in rabbits resembles what has been reported for humans. Rabbits therefore are a relevant model of the human disease caused by type A strains of *F. tularensis*.

## Introduction

*Francisella tularensis*, a gram negative coccobacillus that can cause disease in a number of animals including amoeba, is the causative agent of tularemia [1], [2]. Strains of *F. tularensis* are divided into four subspecies; the most virulent are subspecies *tularensis* (also called type A), followed by *holarctica* (also called type B) [2]. Subspecies *mediasiatica* and *novicida* are not thought to cause human disease. In humans, *F. tularensis* can cause a spectrum of disease that is largely dictated by the route of infection; the most virulent form is pneumonic tularemia, which can cause severe morbidity/mortality at even very low doses as low as 10 CFU [3]. Prior to the introduction of a live, attenuated vaccine, *F. tularensis* was among the most common laboratory-acquired infections [4]. Both the United States (prior to 1969) and the former Soviet Union developed *F. tularensis* as a biological weapon [1], [2]. For these reasons, *F. tularensis* is considered a Category A select agent by the U.S. Department of Health & Human Services [5]. After the anthrax letters in 2001, there was renewed concern that terrorists or rogue nations could be developing biological weapons including tularemia. A vaccine was developed in the 1950's and 60's against tularemia using an attenuated strain of the *holarctica* subtype which is named the live vaccine strain (LVS); LVS provided partial protection of humans and nonhuman primates against aerosol challenge with a type A strain of *F. tularensis* [3], [6]. LVS is available as an Investigational New Drug (IND) for at-risk personnel but is not considered likely to be licensed because the nature of the attenuation is unknown [7]. Nevertheless, efforts are underway to develop and test a new lot of LVS as a potential vaccine, at least for at-risk laboratory personnel [8], [9].

Although cases of tularemia are reported every year, the number of cases is typically quite small and most outbreaks involve the more ubiquitous but less virulent *holarctica* subtype. Outbreaks of pneumonic tularemia involving a type A strain are fairly rare. Human clinical trials to determine efficacy sufficient to support licensure of a vaccine or therapeutic will therefore be ethically and logistically impossible. Licensure of vaccines or therapeutics for protection against *F. tularensis* will only be possible through the use of the FDA's Animal Rule. The Animal Rule requires the use of at least one well-characterized animal model that is relevant to the human disease and in which the mechanism of protection is understood and shown to be similar in humans [10]. It is commonly assumed that to meet the FDA's requirements will require at least two animal models. Mice have been the most extensively used in studies of tularemia; however, there are a number of concerns regarding the use of mice as a model for pneumonic tularemia to support licensure by the Animal Rule. In particular, mice are very susceptible to strains of *F. tularensis*, including strains that are attenuated in humans and nonhuman primates such as *F. novicida* and the live vaccine strain (LVS) [11]. Further, it is difficult to elicit good protection in mouse models against aerosol challenge virulent *F. tularensis* strains at robust challenge doses. These findings suggest that different mechanisms may be involved both in the pathogenesis of *F. tularensis* infection in mice and the protection elicited by vaccines compared to what occurs in humans. Nonhuman primates (NHP) are a relevant model of the human disease and are protected against respiratory challenge with virulent strains of *F. tularensis* after vaccination with LVS. However, they are expensive and difficult to work with in numbers sufficient to generate statistical significance. Particularly for tularemia vaccines, animal models are needed that are more relevant than the mouse to screen candidate vaccines prior to studies with NHP.

Tularemia is also known as rabbit fever and *F. tularensis* was first isolated from a rabbit. An outbreak of pneumonic tularemia in Martha's Vineyards was termed ‘lawnmower tularemia’ after it was revealed that the outbreak resulted from mowing over rabbit dens and possibly infected carcasses [12]. Very little has been done experimentally in rabbits to study the pathology of *F. tularensis* infection or the relevance to the human disease. A British study in the 1970's established that the pathology of experimental pneumonic tularemia in rabbits was similar to that reported in humans and nonhuman primates [13]. A subsequent study found that although LVS was indeed attenuated in rabbits, it was unable to protect the rabbits against an aerosol challenge with a virulent strain of *F. tularensis* [14]. More recently it was reported that LVS was immunogenic in rabbits as seen by the production of a strong antibody response but unfortunately that study did not include a challenge component [15]. Accordingly, these studies were conducted to determine whether rabbits might be a relevant model of the human disease suitable for evaluation of vaccines or therapeutics.

## Results

To develop a rabbit model of pneumonic tularemia, 20 young female NZW rabbits were exposed to aerosols of *F. tularensis* strain SCHU S4 at doses ranging from 190 cfu up to 5.4×10^4^ cfu (Table 1). Rabbits were monitored daily for changes in body temperature, and weight, as well as clinical signs indicative of disease including changes in behavior, anorexia, dehydration, and respiratory distress. Rabbits that became moribund were euthanized promptly, however, as noted in Table 1, 7 of the 20 rabbits were found dead prior to meeting the established criteria for being moribund. To simplify the analysis of the fever and weight loss and to look for trends based on dose, the rabbits were divided into three groups based on dose: low (<3000 cfu; n = 9), medium (3000–9,000 cfu; n = 7) and high (≥10,000 cfu; n = 4). Time to death was extended by 1–2 days for two rabbits in the low dose group relative to other rabbits, giving a longer time to death for the low dose group (5.1 d±1 d) compared to the other dose groups (4.9 d±0.4 d for the medium dose and 4.8 d±0.5 d for the high dose). The difference in time to death between the low dose group and the medium and high dose groups was not statistically significant using a two-tailed Student's *t*-test (*p* = 0.51 and *p* = 0.40, when comparing the low dose group to the medium and high dose groups, respectively).

**Table 1:** Relationship between inhaled dose and disease in rabbits infected with aerosolized F. tularensis.

Inhaled Dose	Max Temp.	Max %	Time to Death	Found Dead or Euthanized?§
		Weight Lost£		
1.9E+02	41.8	−7.7	7	E
7.3E+02	41.1	−10.9	4	E
8.3E+02	41.2	−8.8	5	FD
1.1E+03	40.8	−11.4	5	E
1.1E+03	41.4	−13.9	5	E
1.4E+03	41.7	−12.5	5	E
1.5E+03	41.5	−6.2	4	E
1.8E+03	41.2	−14.0	6	E
2.9E+03	41.5	−4.2	5	FD
3.3E+03	40.3	−6.1	5	E
4.2E+03	41.4	−2.0	4	E
6.6E+03	41	−5.9	5	FD
7.3E+03	41.3	−11.1	5	FD
7.5E+03	41.1	−11.1	5	E
8.4E+03	40.7	−9.1	5	E
8.6E+03	40.6	−9.4	5	FD
1.0E+04	41.1	−2.8	4	FD
1.7E+04	41.5	−6.3	5	E
2.0E+04	40.9	−6.3	5	FD
5.4E+04	41.7	−10.4	5	E

£ Weight lost compared to pre-exposure weight.

§ FD = found dead; E = euthanized when moribund.

**Figure 1:**
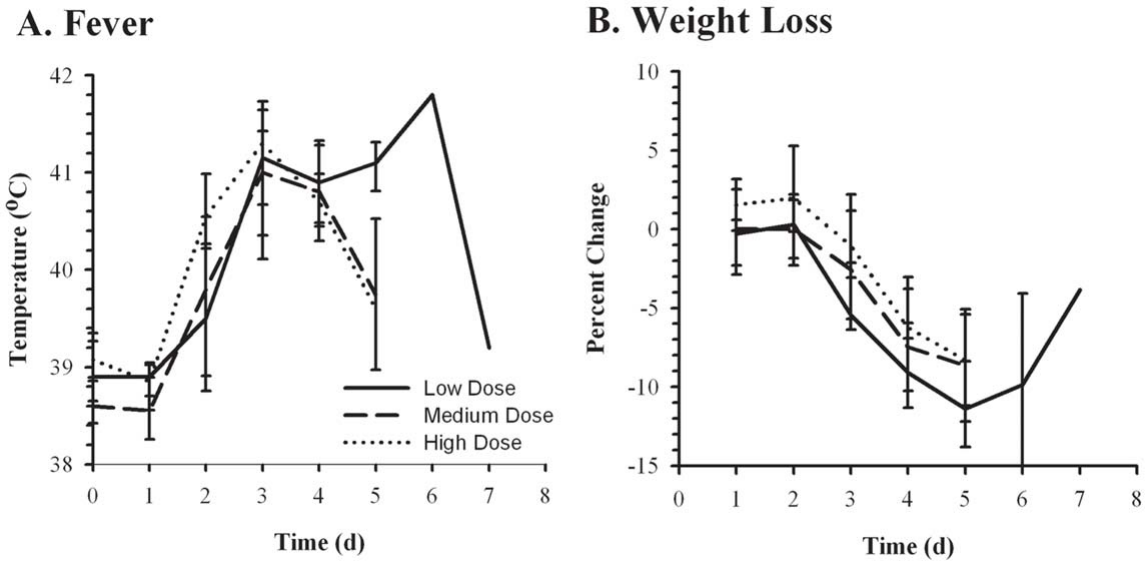
Fever and weight loss after inhalation of F. tularensis. NZW rabbits were exposed to low (<3000 cfu, solid lines), medium (3000–9000 cfu, dashed lines), or high (>9000 cfu, dotted lines) presented doses of F. tularensis SCHU S4 and monitored for changes in body temperature (*A*) and weight (*B*). Graphs show averaged values for each timepoint postexposure with error bars representing the standard deviation.

A febrile response (designated as body temperature >40°C) was first seen in rabbits 2–3 days after exposure (Figure 1A). On average, temperatures remained elevated for 48–72 hours, at which point they began to decline in the medium and higher dose animals. The two rabbits at lower doses that survived out to days 6 and 7 had a persistent fever until the animals succumbed. Table 1 shows the maximum body temperature observed prior to death for all of the rabbits. Regardless of dose, the maximum body temperature observed was similar across the three dose groups (41.3°C, 41.0°C, and 41.3°C for the low, medium, and high doses respectively).

Similarly, weight loss was first detected in rabbits regardless of dose group on day 3 (Figure 1B). Rabbits continued to lose weight until they succumbed to the infection, except for the two rabbits that survived out to days 6 and 7 and which started to recover some weight prior to ultimately succumbing to the infection. The maximum weight loss observed for each rabbit prior to death is shown in Table 1. Animals in the low dose group lost more weight prior to succumbing to the infection (10.7%±2.9% compared to 7.4%±3.3% and 6.4%±3.1% for the medium and high dose groups, respectively). These differences were statistically significant between the low and medium dose groups (*p* = 0.05) but did not rise to the level of significance (*p* = 0.06) between the low and high dose groups.

**Figure 2:**
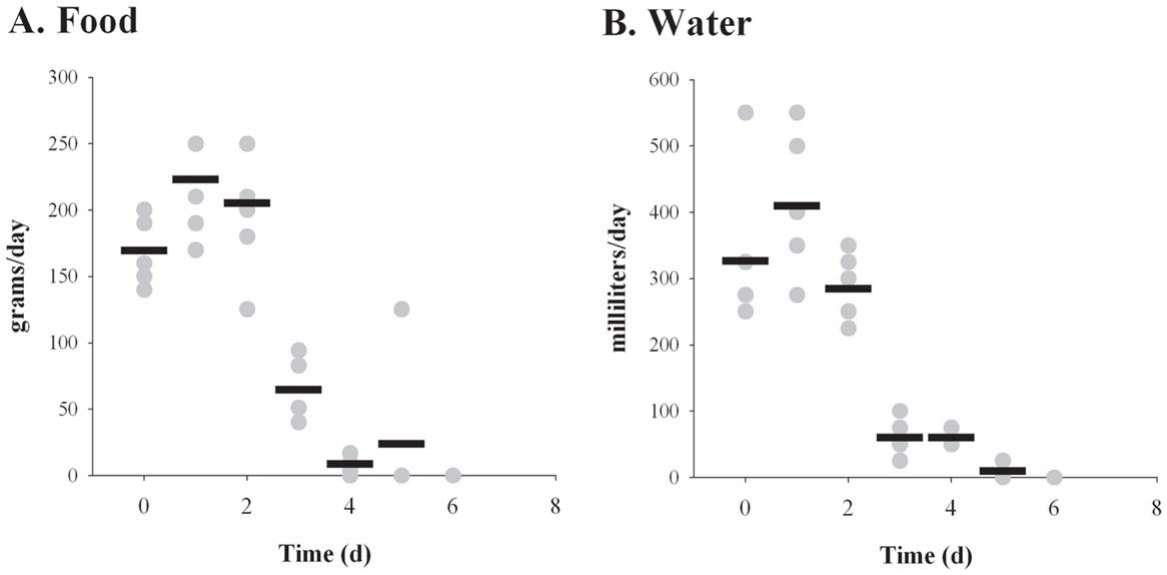
Rabbits reduce food and water intake after inhalation of F. tularensis. Food (*A*) and water (*B*) intake was measured daily for 6 NZW rabbits exposed to aerosolized SCHU S4. Graphs show values for individual rabbits (gray circles) and averaged data (black bars) for each day after exposure.

Six rabbits used in these studies underwent more extensive monitoring to determine other parameters associated with the outcome of pneumonic tularemia. As shown in Figure 2, both food and water intake decreased between days 2 and 3 after exposure, at the same time that fever onset and weight loss was also observed. Intake continued to decline on day 5, with five of the six rabbits consuming no food or water. Due to other clinical signs, those five rabbits were determined to be moribund and subsequently euthanized on day 5; the remaining rabbit did consume some food on day 5 but by day 6 that rabbit had decreased food and water intake to zero. Due to other clinical signs the final rabbit was determined to be moribund on day 6 and was euthanized.

**Figure 3:**
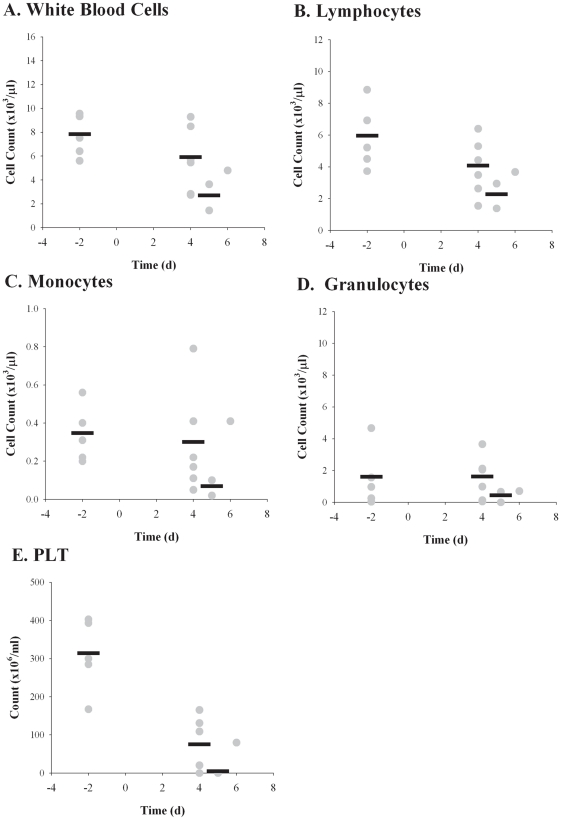
Lymphopenia after inhalation of F. tularensis. Blood samples collected pre- and post-exposure were analyzed for changes in WBC (*A*), lymphocytes (*B*), monocytes (*C*), granulocytes (*D*), and platelets (*E*). Graphs show counts for individual rabbits (gray circles) and averaged data (black bars) for each time point after exposure when blood was drawn (days −2, 2, 4, 6, and at euthanasia).

These six rabbits were also bled to assess hematological changes associated with pneumonic tularemia, beginning two days prior to infection with *F. tularensis*; all of the rabbits were bled on day 4 and then again immediately prior to being euthanized on day 5 (n = 5) or day 6 (n = 1). Bacteremia was assessed by plating on CHA plates. Surprisingly, only one of the six rabbits was positive and that was the rabbit that survived to day 6; it was only positive on day 6 and the levels were very low, 6.1×10^2^ cfu/ml of blood (data not shown). No difference was seen by incubating the blood in 0.1% sodium deoxycholate prior to diluting and plating on CHA. White blood cell counts dropped on day 4 relative to the pre-exposure baseline and then more dramatically on day 5 postexposure (Figure 3A). Further analysis indicated that this decline in WBC was almost entirely due to a drop in the number of mononuclear cells with lymphocytes decreasing on day 4 and lymphocytes and monocytes on day 5. Granulocyte counts were essentially unchanged on day 4 and decreased on day 5. Coagulopathies have not been reported in human cases of tularemia, so it was surprising to see the significant decrease in platelet counts on day 4 compared to pre-exposure baseline levels (*p* = 0.003) (Figure 3E). Of the six animals examined, only one animal had detectable platelets after day 4. Platelets from that animal had an elevated MPV on day 6 (11) compared to day 4 (7.7) which is suggestive of a release of immature platelets from the bone marrow. The other hematological parameters evaluated (RBC, HGB, HCT, MCV, MCH, MCHC, RDWc, PCT, and PDWc) showed little or no change after exposure (data not shown).

**Figure 4:**
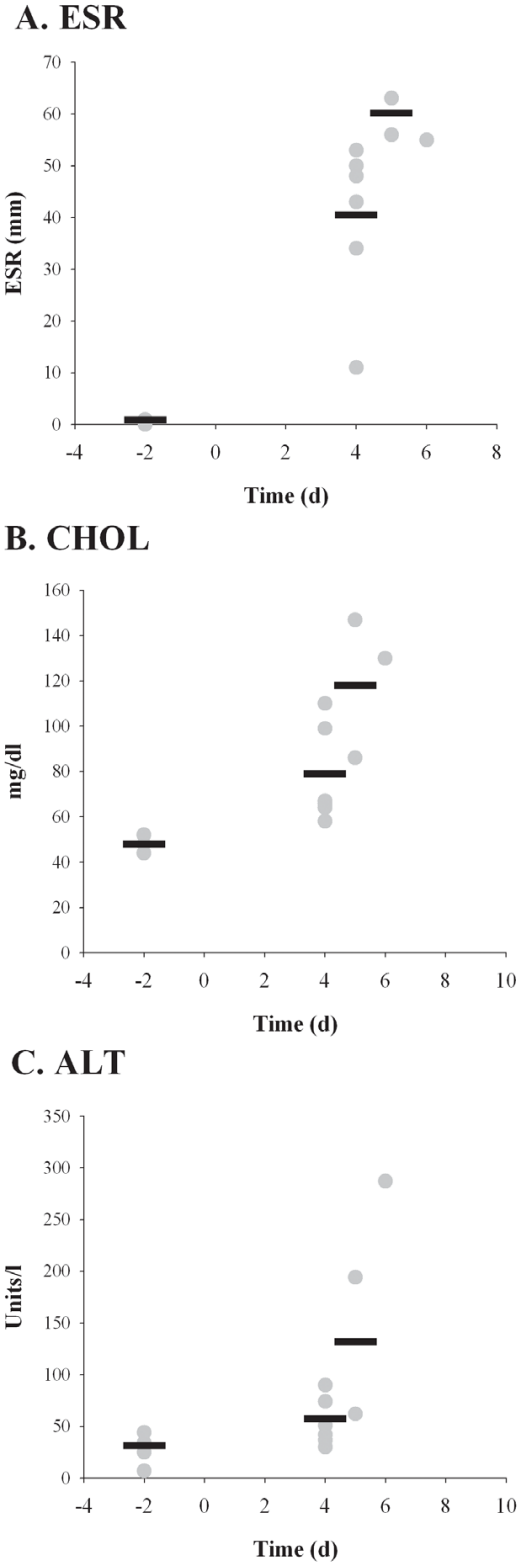
Increases in erythrocyte sedimentation rate and liver function markers after inhalation of F. tularensis. Blood samples collected pre- and post-exposure were analyzed for changes in ESR (*A*), cholesterol level (*B*), and ALT (*C*). Graphs show values for individual rabbits (gray circles) and averaged data (black bars) for each time point after exposure when blood was drawn (days −2, 2, 4, 6, and at euthanasia).

Erythrocyte sedimentation rate (ESR) was performed on blood samples as a non-specific measure of inflammation and C-reactive protein levels (Figure 4A). Pre-exposure ESR levels were between 0 and 1 mm, as expected. ESR levels were increased substantially on day 4, ranging from 11 to 53, with the average 40. Not surprisingly, this difference was statistically significant from the pre-exposure baseline (*p* = 0.002). The ESR average increased still further, peaking on day 5 at 60 mm. Similarly, serum cholesterol and alanine aminotransferase (ALT) levels were elevated on day 4 and even more noticeably on day 5 (Figure 4 B and C); these differences were also significant compared to pre-exposure baseline levels (*p* = 0.05 and *p* = 0.02 for ALT and cholesterol, respectively). Other liver parameters (ALP, GGT, BA, TBIL, ALB, and BUN) evaluated did not show remarkable or consistent differences between baseline and post-exposure blood samples in all animals (data not shown).

**Figure 5:**
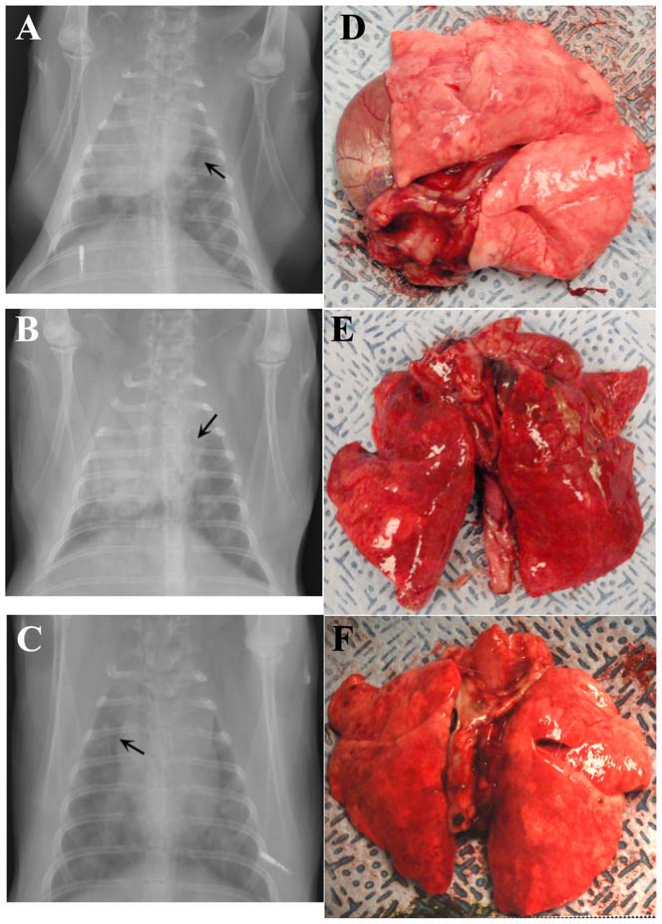
Radiographs and gross pathological changes in lungs demonstrating development of pneumonic tularemia in rabbits. Anterioposterior radiographs (A–C) taken of three anesthetized rabbits four days after infection with *F. tularensis* illustrating development of bilaterial pneumonia. Arrows on radiographs indicate presence of air bronchograms. Pictures of the lungs removed from those same three rabbits at necropsy on day 5 (*D,E*) or day 6 (*F*) after infection.

**Figure 6:**
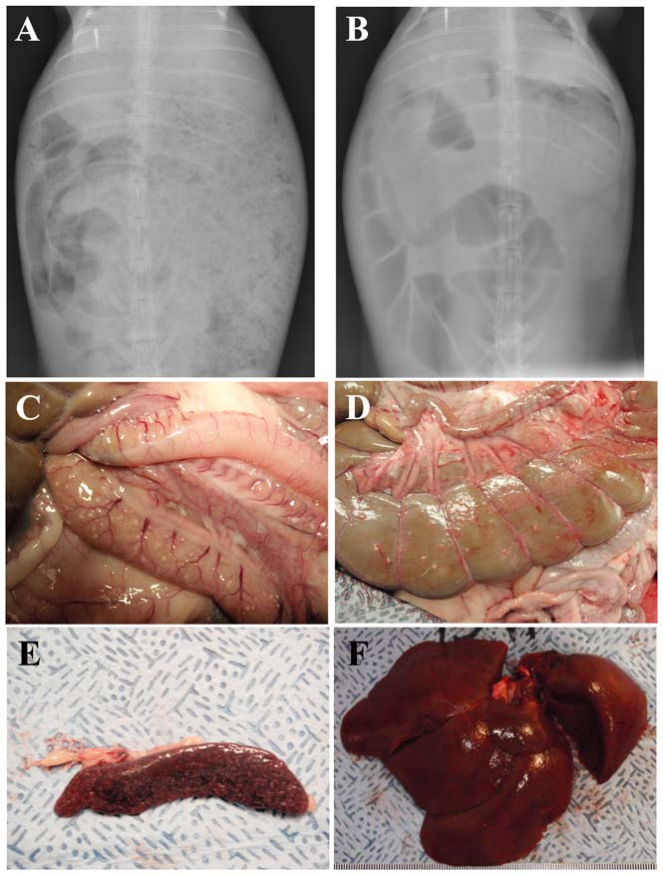
Gastrointestinal pathology and hepatosplenomegaly associated with tularemia in rabbits. Anterioposterior radiographs showing the abdomen of a single representative rabbit two days prior (*A*) and four days after (*B*) infection with F. tularensis, illustrating gas production and distension of the intestines and stomach. Pictures of the small intestines (*C*), colon (*D*), spleen (*E*) and liver (*F*) examined at necropsy from a single representative rabbit.

The progression of pneumonic tularemia in the rabbits was also assessed by x-rays and gross pathological evaluation of euthanized rabbits. Figure 5 shows the chest x-rays and corresponding gross lung pathology for three representative rabbits taken on 4 (Figure 5A & B) or 6 (Figure 5C) days after infection. Prominent in all three images are areas of parenchymal consolidation and loss of lung volume: Figure 5A shows collapse of right upper lobe with upward expansion of right lower lobe. The x-rays images of the lung show areas of patchy (best exemplified in Figures 5B and C) parenchymal consolidation and prominent air bronchograms are present suggesting the presence of pneumonia. As a result of these abnormalities, the margins of the heart and diaphragm are erased instead of clear. Supporting these findings, the lungs removed from these animals show numerous distinct areas of hemorrhage (Figure 5E) and subpleural areas of nodular pearl like consolidation (Figure 5D & 5F).

Radiographs of the abdominal regions show considerable changes 4 days after infection, including abnormal gas distribution with distension of the stomach as well as of the small and large intestines (Figure 6, A & B) suggesting the presence of ileus. Pictures taken at necropsy confirm the gastric distension and evidenced the presence of multiple, small white foci in the small (Figure 6C) and large intestines where they were closely associated with hemorrhage of the serosa (Figure 6D). Similar white foci were also seen in the kidneys (data not shown). All necropsied rabbits showed evidence of hepatosplenomegaly; the weight of the liver and spleen relative to the body weight of the infected rabbits (3.3% and 0.12%, respectively) was nearly 50% more than for a healthy female rabbit of equivalent age [16]. Spleens from infected rabbits show extensive necrosis and were darker in color than normal spleens and demonstrated extensive white small nodules (Figure 6E). Livers were smooth in appearance but not coloration, with a mottled pattern of dark and light foci (Figure 6F).

**Figure 7:**
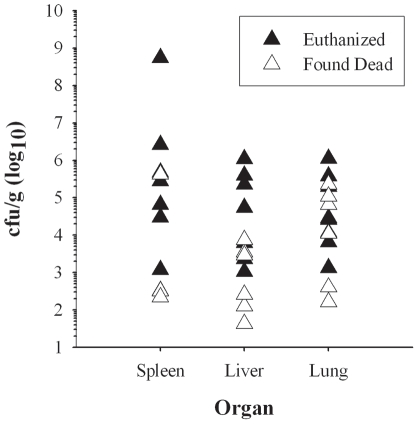
Bacterial load in organs of rabbits that succumbed to pneumonic tularemia. Samples of the spleen, liver, and lungs were homogenized, diluted in PBS and plated on CHA to determine bacterial load in rabbits. Graph shows log10 data for individual rabbits that were either euthanized (filled triangles) or found dead (open triangles).

Samples of liver, spleen and lung taken at necropsy were homogenized and dilutions plated to determine the extent of bacterial dissemination to these tissues; the results are shown in Figure 7 plotted on a log10 scale. The concentration of bacteria in all three tissues was highly variable between individual rabbits, particularly in the spleen. In both the spleen and liver, the concentration of bacteria was 2–3 logs higher in rabbits that were euthanized compared to those that were found dead. In the spleen, the average was 7.8×10^7^ cfu/g for euthanized rabbits as compared to an average of 6.0×10^4^ cfu/g for rabbits that were found dead. In spleens from three of the animals found dead no counts were obtained. In the liver the average was 2.5×10^5^ cfu/g in euthanized rabbits compared to 2.1×10^3^ in rabbits found dead. In the lungs, in contrast, there was considerably more overlap between concentrations from rabbits that were euthanized or found dead; the average cfu/g from the lungs were 2.5×10^5^ for euthanized rabbits and 6.0×10^4^ for rabbits found dead. However, because of the variability in the concentration of *F. tularensis*, none of the differences were statistically significant between rabbits that were euthanized or found dead for any of the organs assayed.

## Discussion

The data presented here detail the disease course and outcome of pneumonic tularemia following infection with type A strain SCHU S4 in New Zealand White rabbits, a potential model of the human disease. The value of the current experimental model is predicated on its ability to reproduce the key patho-physiological events of the human disease. Thus, our findings here for the rabbit model match well with what has been reported in human cases of pneumonic tularemia, including fever, weight loss, elevations in ALT and ESR, leukopenia, and radiographs detailing the development of bibasilar pneumonia [1], [3], [12], [17]. Radiographs demonstrating the excess gas distension of the gastrointestinal system in the rabbits could correspond with reports in humans of abdominal pain, diarrhea, and anorexia. In keeping with this observation, we noted a pronounced decrease in food and water intake by the rabbits at the same time that the initial clinical signs were reported. Ileus has not been reported in humans infected with tularemia and is likely a result of the cessation of eating and drinking in rabbits. Whether it is important in the outcome of the infection in rabbits is not clear and further work is needed to determine whether this is a critical difference in the pathophysiology of tularemia in rabbits and humans. Necropsy findings supported the x-ray findings in both the lungs and intestines and also demonstrated widespread dissemination of the bacteria and hepatosplenomegaly in the rabbits which has been reported in human tularemia [18]. The gross pathology of the lungs suggest at least two different processes in the rabbits, one with nodular lesions and one more hemorrhagic. The differences seen do not appear to be a result of dose or time to death. It is unclear why we are seeing multiple processes in the lung, however, it should be noted that NZW rabbits are not completely inbred so this may be partly due to genetic differences between rabbits.

Although *F. tularensis* was first isolated from a rabbit and tularemia is also known as ‘rabbit fever’, there have been few studies detailing experimental infection with *F. tularensis* in rabbits, particularly for infection via the respiratory tract. Baskerville & Hambleton examined the pathogenesis of pneumonic tularemia in New Zealand White rabbits after exposure to aerosolized *F. tularensis* strain SCHU S4 [13]. These investigators reported calculated inhaled retention doses between 2×10^4^–4×10^8^ cfu; however, they did not report how the ‘calculated inhaled retention dose’ was calculated. Our doses are calculated as presented doses, which are represented as the total amount of bacteria inhaled [19]. The retained dose typically represents the amount of bacteria which deposit in the lung and are not cleared by the mucociliary escalator or other mechanisms, and as such is some fraction of the presented dose. Therefore the doses reported by Baskerville & Hambleton are at least 1–4 log_10_ cfu higher than the challenge doses we delivered in these studies. Importantly, our study demonstrates that rabbits are susceptible to very low doses of tularemia without appreciable increases in time to death. Over the course of the study Baskerville & Hambleton noted pathological changes first in the lung, progressing into the cervical and bronchial lymph nodes, the liver, and the spleen. Similar to the results reported here, they noted elevations in body temperature on day 2, fever >40.2°C by day 3, and a reduction in food intake. A subsequent publication reported leukopenia and an increase in neutral fats 2–3 days after infection [14]. In the current report we were able to determine that the major effect of the infection on white blood cells is due to a depletion of lymphocytes. Platelet counts were also depleted over the course of the infection. Therefore, previous reports, coupled with our current data, strongly suggest that inhalation of *F. tularensis* in rabbits constitutes a relevant model of the human disease. Further work is needed, including detailed pathogenesis and immunological studies, to elucidate the pathophysiology of tularemia in rabbits to aid in determining whether rabbits are a suitable model to meet the requirements of the Animal Rule.

An additional requirement of the FDA's Animal Rule for selection of animal models to use in pivotal efficacy studies is that the mechanism of amelioration/protection must be the same in the animal model as in the human. While ethical and logistical reasons impede the ability to conduct an efficacy study of medical countermeasures against pneumonic tularemia in humans, there exists data from studies conducted in the 1950's and 1960's that can be used to guide the selection of an animal model. In particular, vaccination with the live vaccine strain (LVS) of *F. tularensis* by the intracutaneous or aerosol routes induced at least partial protection in humans against aerosol challenge with the SCHU S4 strain of *F. tularensis*; humans were protected against aerosol challenge with 200–2,000 cfu but not 10-fold higher doses [3], [20], [21], [22]. A prior report has indicated that vaccination with LVS increased time to death of rabbits challenged with SCHU S4 (at a dose of 5×10^5^ cfu ) but no rabbits survived challenge [14]. In ongoing studies we have found similar results: vaccination with LVS extended time to death typically by 1–2 days but no LVS-vaccinated rabbits survived aerosol challenge with SCHU S4 at doses between 1,000–10,000 cfu [23]. In contrast, some rabbits vaccinated with attenuated mutants of SCHU S4 did survive aerosol challenge. We believe therefore that the rabbit model will prove to be a more stringent model of protection than nonhuman primates or rats but not as rigorous as the mouse model. However, this will need to be further evaluated before the rabbit can be accepted as a suitable model for tularemia vaccine efficacy studies.

The animal models most commonly used to study pneumonic tularemia are mice and nonhuman primates (NHP). Many aspects of the human disease are recapitulated in these models. There are concerns about the use of the mouse model as mice are susceptible to strains of *F. tularensis* that are attenuated in NHP and humans and vaccination with LVS poorly protects against respiratory challenge with *F. tularensis* [11]. The susceptibility to attenuated strains suggests that different mechanisms are involved in the pathogenesis of *Francisella* infection in mice relative to humans or other species. Nonhuman primates have generally been considered good models of human tularemia; however, the FDA's Animal Rule has commonly been interpreted to mean that testing in more than one animal model is necessary to satisfy the FDA's requirements for pivotal efficacy studies to support vaccine licensure. There have also been recent reports detailing tularemia studies in rats [24], [25], [26]. Similar to what we report here and prior reports on rabbits, rats have similar pathology to what has been reported in humans and are resistant to inoculation with LVS. Therefore, there is a need to explore both rats and rabbits as alternative models of pneumonic tularemia to comprehensively test the efficacy of vaccines against this disease.

The current work suggests that rabbits also constitute a valuable model as they reproduce the key pathophysiological aspects of the human disease. In addition to showing similar gross pathology to humans infected with virulent *F. tularensis*, rabbits are resistant to LVS [14], [15]. However, although vaccination of rabbits with LVSlengthened survival of these animals relative to unvaccinated controls after aerosol challenge with SCHU S4 (at a high dose) it does not prevent death [14]. Thus, further studies characterizing the immunologic response of rabbits to *F. tularensis* will be necessary to definitively establish the value of rabbits as a model for vaccine efficacy in pneumonic tularemia.

## Materials and Methods

### Rabbits

Young female New Zealand White (NZW) rabbits were obtained from Myrtle's Rabbitry (Thompsons Station, TN) and housed in the University of Pittsburgh Regional Biocontainment Laboratory (RBL) at ABSL-3 for the duration of the studies. Prior to exposure, IPTT-300 temperature/ID chips (BioMedic Data Systems, Seaford, DE) were implanted subcutaneously. Temperature was read using a DAS-7000 reader (BioMedic Data Systems). Food and water intake was monitored and recorded twice daily. All studies were approved by the University of Pittsburgh's Institutional Animal Care and Use Committee, protocol number 1004196. The University of Pittsburgh is an AALAC-approved facility.

### Bacteria

Virulent *F. tularensis* strain SCHU S4 was used in these studies. A stock was prepared by one *in vitro* passage of an isolate obtained from Dynport Vaccine Company (Frederick, MD). This stock was washed one time and frozen at ^−^80°C in Brain Heart Infusion broth (BHI) containing 20% glycerol. For aerosol exposures, SCHU S4 was streaked on cysteine heart agar (CHA) containing 1% hemoglobin and incubated for 2 days at 37°C in 5% CO_2_. Individual colonies were picked off the plate and resuspended in PBS to an OD_600_ reading of 0.1; 0.5 ml of diluted SCHU S4 was added to a baffled polycarbonate vented 125 ml flask containing 24.5 ml of BHI. The flask was placed in a shaking incubator and incubated overnight at 37°C. At approximately 18 h after the culture was started, it was removed from the shaker and the OD read at 600 nm. Using a growth curve from prior studies, the concentration was adjusted in BHI to the concentration desired for aerosol exposures. During preparation and throughout the exposures, *F. tularensis* was kept on ice. After the exposures were completed, samples of the nebulizer and all-glass impinger (AGI) contents were diluted 10-fold in PBS, spread on quad CHA plates in duplicate and incubated at 37°C 5% CO_2_ for 2 days prior to counting.

### Aerosols

Aerosols were conducted inside a class III biological safety cabinet (Baker Co., Sanford, ME) located inside the aerobiology suite of the University of Pittsburgh Regional Biocontainment Laboratory (RBL). Animals are transported from their holding room to the aerobiology suite via mobile transfer carts (Baker). Rabbits were exposed two at a time for 10 minutes in a nose-only exposure chamber (CH Technologies, Westwood, NJ) while plethysmography data was collected in real-time using Buxco XA software (Buxco Research Systems, Wilmington, NC) during the exposure. Aerosols were created with a vertical discharge 3-jet Collison nebulizer controlled by the AeroMP aerosol management platform (Biaera Technologies, Hagerstown, MD). Aerosol samples were collected using an all-glass impinger (AGI) containing 10 ml of BHI and 0.001% Antifoam A (Sigma-Aldrich, St. Louis, MO) operating at 6 lpm for the duration of the exposure. At the conclusion of the aerosol, the system was purged with clean air for 5 minutes after which the rabbits were transported back to their holding room. Aerosol concentration was determined was described previously: the bacterial count measured in the AGI was multiplied by the volume of liquid in the AGI which was then divided by the product of the flow of air through the AGI and the duration of the exposure [19]. Inhaled (presented) dose was also determined as described previously: the aerosol concentration multiplied by the rabbit's minute volume and the duration of the exposure [19].

### Phlebotomy and X-rays

Rabbits were anesthetized first by subcutaneous injection of ketamine (80 mg/kg) and xylazine (8 mg/kg). Isoflurane (dose) was used to maintain anesthesia while the rabbit was bled via the ear vein. Approximately 2 ml of blood was collected per procedure; a portion was used for clinical chemistries, for bacteremia, and for erythrocyte sedimentation rate, and for measurement of antibody titers. Once phlebotomy was completed the rabbit was laid prone and lateral and chest digital x-rays were taken (Kodak). The xylazine was reversed by i.m. injection of dose (mg/kg) yohimibine. The rabbit was returned to its cage and monitored until it recovered from anesthesia.

### Monitoring

Rabbits were monitored twice daily after exposure. Body weight was recorded once in the morning and body temperature was recorded twice daily. A clinical scoresheet was used to record body temperature and changes in weight, behavior and appearance. Scores were recorded for four criteria at least once daily. Temperature: >34°C and <40°C = 1; >40 = 2; >41 = 3; >42 = 4; >32 & <34 = 5; <32 = euthanize promptly. Weight Loss: up to 10% = 1; 11–15% = 2; 16–20% = 3; 21–25% = 4; >25% euthanize promptly. Appearance: Normal = 1; reduced grooming = 2; ruffled fur = 3; hunched = 4; respiratory distress = 5. Behavior: Normal = 1; less peer interaction = 2; huddled = 3; moving only when prodded = 4; no response = 5. Monitoring was increased to every six hours if an animal's score was 12 or higher and if the score reached 15 or higher the rabbit was considered moribund and was euthanized promptly. Food and water intake was recorded separately. When rabbits reached a score at which they were considered to be moribund, they were promptly euthanized.

### Euthanasia and necropsy

Rabbits were euthanized by intravenous injection of sodium pentobarbital at 100 mg/kg. Once death was confirmed rabbits were necropsied. Pictures were taken of organs removed from the rabbits and samples of the liver, spleen and lung were taken for determining bacterial loads.

### Erythrocyte Sedimentation Rates

Rabbit whole blood collected in EDTA was pipetted using a glass Pasteur pipet into glass Wintrobe tubes. After one hour, the degree of sedimentation was recorded in mm for each rabbit.

### Hematology

Rabbit whole blood collected in EDTA was analyzed on a VetScan HM2 (Abaxis, Union City, CA) to determine the complete blood count and on a VetScan VS2 (Abaxis) to measure blood chemistry, using a profile optimized for mammalian liver function.

### Statistical methods

Data was collected and organized using spreadsheets in Microsoft Excel 2007; analysis was conducted using a two-tailed Student's t-test available in the Analysis Toolpak add-in.
